# Role of the Lateral Paragigantocellular Nucleus in the Network of Paradoxical (REM) Sleep: An Electrophysiological and Anatomical Study in the Rat

**DOI:** 10.1371/journal.pone.0028724

**Published:** 2012-01-03

**Authors:** Chrystelle Sirieix, Damien Gervasoni, Pierre-Hervé Luppi, Lucienne Léger

**Affiliations:** 1 Sleep Team, Lyon Neuroscience Research Center, Lyon, France; 2 Unité Mixte de Recherche 5292, Centre National de la Recherche Scientifique, Lyon, France; 3 Unité 1028, Institut National de la Santé et de la Recherche Médicale, Lyon, France; 4 University Lyon 1, Lyon, France; The Research Center of Neurobiology-Neurophysiology of Marseille, France

## Abstract

The lateral paragigantocellular nucleus (LPGi) is located in the ventrolateral medulla and is known as a sympathoexcitatory area involved in the control of blood pressure. In recent experiments, we showed that the LPGi contains a large number of neurons activated during PS hypersomnia following a selective deprivation. Among these neurons, more than two-thirds are GABAergic and more than one fourth send efferent fibers to the wake-active locus coeruleus nucleus. To get more insight into the role of the LPGi in PS regulation, we combined an electrophysiological and anatomical approach in the rat, using extracellular recordings in the head-restrained model and injections of tracers followed by the immunohistochemical detection of Fos in control, PS-deprived and PS-recovery animals. With the head-restrained preparation, we showed that the LPGi contains neurons specifically active during PS (PS-On neurons), neurons inactive during PS (PS-Off neurons) and neurons indifferent to the sleep-waking cycle. After injection of CTb in the facial nucleus, the neurons of which are hyperpolarized during PS, the largest population of Fos/CTb neurons visualized in the medulla in the PS-recovery condition was observed in the LPGi. After injection of CTb in the LPGi itself and PS-recovery, the nucleus containing the highest number of Fos/CTb neurons, moreover bilaterally, was the sublaterodorsal nucleus (SLD). The SLD is known as the pontine executive PS area and triggers PS through glutamatergic neurons. We propose that, during PS, the LPGi is strongly excited by the SLD and hyperpolarizes the motoneurons of the facial nucleus in addition to local and locus coeruleus PS-Off neurons, and by this means contributes to PS genesis.

## Introduction

Since the discovery of the second state of sleep named paradoxical (or REM) sleep (PS) fifty years ago, vigorous efforts have been accomplished to decipher the neuronal network responsible for its genesis and maintenance [1], [2], [3]. It is now well accepted that this genesis occurs in the brainstem [4], [5], [6] and involves a true network of distributed groups of neurons ([3], [7], [8].

Recent studies have suggested that the lateral paragigantocellular nucleus (LPGi) [9], otherwise defined as the rostral ventrolateral medulla and known as a sympathoexcitatory site regulating blood pressure, [10], [11] could be an integral part of the network regulating PS. Indeed, this nucleus contains a large number of neurons expressing Fos, a marker of neuronal activation, during the PS hypersomnia that follows a selective deprivation [12], [13]. Among these Fos-labelled neurons, more than two-thirds are GABAergic [14] and more than one fourth send efferent fibers to the wake-active locus coeruleus nucleus (LC) [12], [13]. Since the LC neurons are inhibited by GABA during PS [15], it has been postulated that this inhibition could partly arise from the LPGi [13]. In addition, the LPGi contains catecholaminergic neurons that express Fos during PS deprivation and, consequently, could be active during waking (W) and participate in the inhibition of PS [16]. Given these data, it can be hypothetized that the LPGi contains neurons selectively active during PS (PS-On neurons) [17] and neurons silent during PS (PS-Off neurons), However, their presence remains to be demonstrated *in vivo*.

Knowing that the LPGi sends efferent, likely inhibitory axons to the LC provides only one piece of information regarding its role in the network regulating PS. Other central targets of the LPGi have been described, e.g. the solitary tract, parabrachial and Kölliker-Fuse nuclei [18]. Together with the descending projections to the spinal cord, these projections are known to be involved in the control of the cardiovascular system [11], [19], [20] but nothing is known of their potential role in the regulation of PS.

Information is also lacking as regards the localization of the neurons that activate the LPGi during PS. Several anatomical studies, using retrograde or anterograde tracers, have disclosed multiple central origins for the axons innervating the LPGi [21], [22], [23], [24]. Among these areas, several contain neurons active during PS, as seen with electrophysiological techniques or the immunohistochemical detection of Fos [12], [14], [25], [26], [27]. However, it is not known which, among these active cells, may influence the LPGi during PS.

To get more insight into the anatomical place of the LPGi and be able to suggest possible functional role(s) for this nucleus in the PS regulatory network, we first performed extracellular recordings of LPGi neurons across the sleep-waking cycle using the head-restrained rat model [15], [28]. Secondly, facing the relative paucity of the literature concerning the efferent projections of the LPGi apart from those of catecholaminergic nature and related to cardiovascular control [18], [29], [30], we injected *Phaseolus vulgaris* leucoagglutinin (PHA-L) in this nucleus. Thirdly, to visualize the areas potentially controlling the LPGi during PS, we combined injections of a retrograde tracer (cholera toxin, subunit B (CTb)) in the LPGi with the detection of Fos generated during a PS deprivation-recovery protocol (see [13].

Our results show that the LPGi contains both PS-On and PS-Off neurons. In addition, they suggest that the LPGi is strongly controlled by the sublaterodorsal nucleus (SLD), recognized as the PS executive area. Finally, the LPGi is likely involved in the inhibition of the facial motoneurons that occurs during PS.

## Results

### Electrophysiological study

In order to characterize the mode of discharge of the LPGi neurons across the vigilance states, a sample of 50 neurons was recorded in the LPGi (n = 10 rats) during at least one complete sleep-waking cycle (with W, slow-wave sleep (SWS) and PS). The localization of these neurons was estimated post-mortem by assessing on brainstem sections the location of the tracer deposits. All neurons were located in the main part of the LPGi, caudally to the facial nucleus.

**Figure 1:**
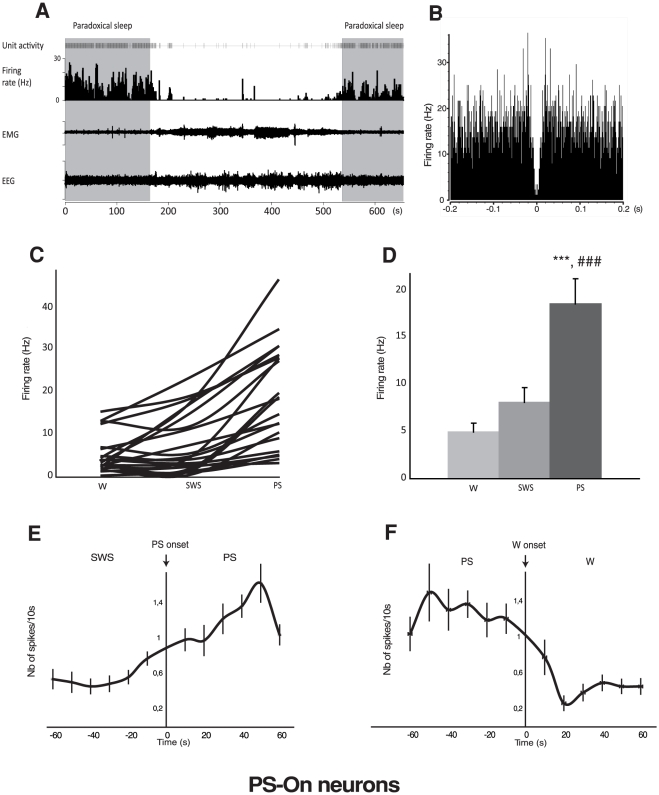
Discharge of the LPGi PS-On neurons across the sleep-waking cycle. A. Unit activity of a representative PS-On neuron across 2 PS episodes bordering Waking and SWS episodes. It is clearly seen that this neuron displays a higher rate of activity during PS and that this activity starts before and ends after the PS episode. B. Autocorrelogramm of another PS-On neuron. The absence of peaks is indicative of the absence of bursting. C. Firing rate of the 21 PS-On neurons recorded in the LPGi across the sleep-waking cycle. D. Firing rate of the PS-On neurons recorded in the LPGi in the 3 vigilance states. Data are expressed as mean ± SEM, hellohellohello P≤0.001 vs Waking, ### P≤0.001 vs SWS. E. Number of spikes per 10s during the SWS/PS transition. The majority of the PS-On neurons start to significantly increase their firing rate about 10 s before the onset of PS. F. Number of spikes per 10s during the PS/W transition. The PS-On neurons stop discharging a few s after the end of the PS episodes.

Among the 50 neurons, 21 could be classified as PS-On neurons (Table 1, Fig. 1). Indeed, their firing rate was significantly increased during PS vs. SWS (18.61±2.64 Hz vs 8.11±1.61 Hz, p<0.001) and vs W (18.61±2.64 Hz vs. 4.94±0.99 Hz, p<0.001). Their mean firing rate was not significantly different between W and SWS but it is interesting to note that about half of them were almost completely silent during both these states (Fig. 1C). The value of the coefficient of variation of the PS-On group assessed that their firing mode was more phasic during W (4.08±1.03) than during SWS (2.28±0.31) and PS (1.98±0.11). At close observation of the traces during PS, it can be seen that the PS-On neurons display a tonic mode of discharge but with obvious phasic increases of activity (Fig. 1A). However, no sign of rhythmicity was detected in the autocorrelograms (see the absence of regular peaks in Fig 1B). No differences were noticed for the asymmetry index across vigilance states (W = 0.30±0.06; SWS = 0.28±0.06; PS = 0.26±0.05) (Table 1). The low value of this index is indicative of an irregular mode of discharge during all states. In addition and of functional significance, the PS-On neurons started to significantly increase their firing rate about 10 s before the onset of PS (Fig. 1A,E). They stopped firing about 20 s after the end of PS (Fig. 1A,F).

**Table 1:** Electrophysiological characteristics of the three types of neurons recorded in the LPGi.

	Firing rate (Hz)			Asymetric index			Coefficient of variation		
	Wake	Slow wave sleep	Paradoxical sleep	Wake	Slow wave sleep	Paradoxical sleep	Wake	Slow wave sleep	Paradoxical sleep
**PS-On neurons**	4.94±0.99	8.11±1.61	18.61±2.64^***,^ ###	0.30±0.06	0.28±0.06	0.26±0.05	4.08±1.03	2.28±0.31°	1.98±0.11^**^
**PS-Off neurons**	5.72±1.57	4.36±1.45	0.45±0.14^*,^ #	0.35±0.09	0.40±0.08	N/A	2.90±0.32	2.81±0.67	N/A
**State-indifferent neurons**	13.08±3.66	13.15±3.21	13.72±3.81	0.33±0.06	0.39±0.07	0.25±0.05^*##^	3.85±0.49	3.50±1.01	2.76±0.65^**^

Data obtained in 21 PS-On, 8 PS-Off and 21 state-indifferent neurons. Figures refer to mean ± S.E.M. N/A, not available, hello P≤0.05, hellohello P≤0.01, hellohellohello P≤0.001 vs Waking;

# P≤0.05,

## P≤0.01,

### P≤0.001 vs SlowWaveSleep;

° P≤0.05 vs Waking.

**Figure 2:**
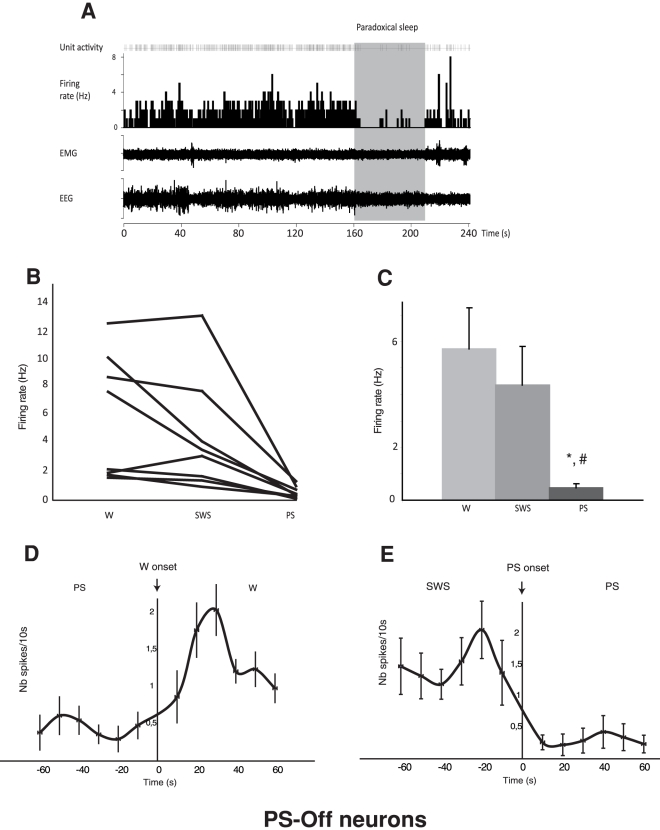
Discharge of the LPGi PS-Off neurons across the sleep-waking cycle. A. Unit activity of a representative PS-Off neuron across a PS episode following SWS. This neuron ceases its discharge immediately after the initiation of the PS episode and recovers at the end of the PS episode. B. Firing rate of the 8 PS-Off neurons recorded in the LPGi across sleep-waking cycle. C. Firing rate of the PS-Off neurons recorded in the LPGi in the 3 vigilance states. Data are expressed as mean ± SEM, hello P≤0.05 vs SWS, # P≤0.05 vs PS. D. Number of spikes per 10s during the PS/W transition. E. Number of spikes during the SWS/PS transition. The majority of the PS-Off neurons stop firing immediately after the onset of PS.

The second group was formed by 8 neurons that were considered as PS-Off neurons (Fig. 2). Indeed, their firing rate was significantly decreased during PS vs W (0.45±0.14 Hz vs. 5.72±1.57 Hz, p<0.05) and vs. SWS (0.45±0.14 Hz and 4.36±1.45 Hz, p<0.05). These neurons stopped firing immediately at the onset of the PS episodes and showed a non-significant difference of their firing rate between W and SWS (Table 1, Fig. 2B,C). However, when looking at the individual discharge rates, it was obvious that this population was heterogeneous, in spite of its small size. Whereas most neurons did not change their mean firing rate from W to SWS, 2 neurons in our sample decreased their rate of discharge during this transition (Fig. 2B). As an average, the value of the coefficient of variation of the PS-Off neurons was indicative of a rather tonic firing rate during both W (2.90± 0.32) and SWS (2.81±0.67). The low value of the asymmetry index during both W (0.35±0.09) and SWS (0.40±0.08) indicated an irregular mode of discharge (Table 1).

The last group (21 cells) was formed by neurons that did not show a state-related activity. Indeed, their mean firing rate was not significantly different during W (13.08 ±3.66 Hz), SWS (13.15±3.21 Hz) and PS (13.72 ± 3.81 Hz) (Table 1). These state-indifferent neurons exhibited a somewhat irregular mode of discharge during the sleep-waking cycle (value of the asymmetry index between 0.25±0.05 and 0.39±0.07).

**Figure 3:**
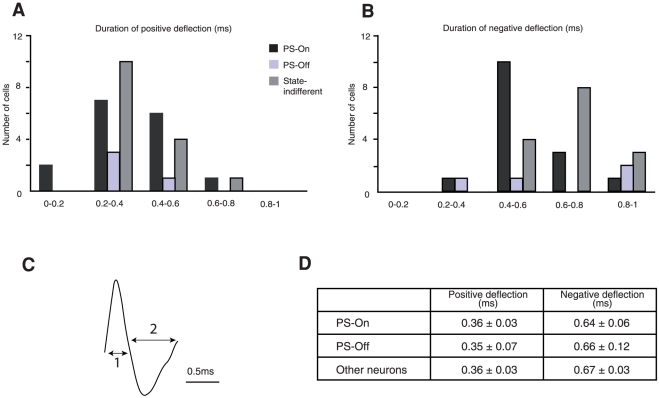
Spike duration of the PS-On, PS-Off and state-indifferent neurons recorded in the LPGi. A,B. Duration of the positive and negative deflections. C. Typical example of a PS-On neuron extracellular waveform (1 corresponds to the length of the positive deflection and 2 to the length of the negative deflection). D. Duration of the positive and negative deflections of the spikes of all recorded neurons.

Finally, we determined whether the three groups of neurons recorded in the LPGi could be differentiated by their spike characteristics (i.e. spike waveform and duration). The extracellular action potentials are usually recorded as a positive deflection followed by a negative one. According to a previous classification for brainstem neurons [31], spikes with a positive deflection lasting more than 0.75 ms followed by long (> 1ms) negative deflection are classified as broad spikes. Spikes with shorter characteristics are classified as brief spikes. Here, all subgroups of neurons displayed positive deflections around 0.36 ms and negative deflections around 0.66 ms, i.e. a total spike duration close to 1 ms (Fig. 3). According to both values, all neurons presented brief spikes, therefore could not be differentiated on this ground.

### Neuroanatomical study

In all rats used in the anatomical study, the flowerpot method was efficient in depriving the animals of PS and subsequently inducing a PS hypersomnia, similarly to the previous descriptions from authors using this technique [12], [13], [32]. Indeed, during the 150 min preceding the euthanasia, the amount of PS was significantly different among the PS-control (PSC), PS-deprived (PSD) and PS-recovery (PSR) groups. It constituted 11.2% of the time in the PSC group, 1.1% in the PSD group and 28.7% in the PSR group.

#### Efferent projections of the LPGi

**Figure 4:**
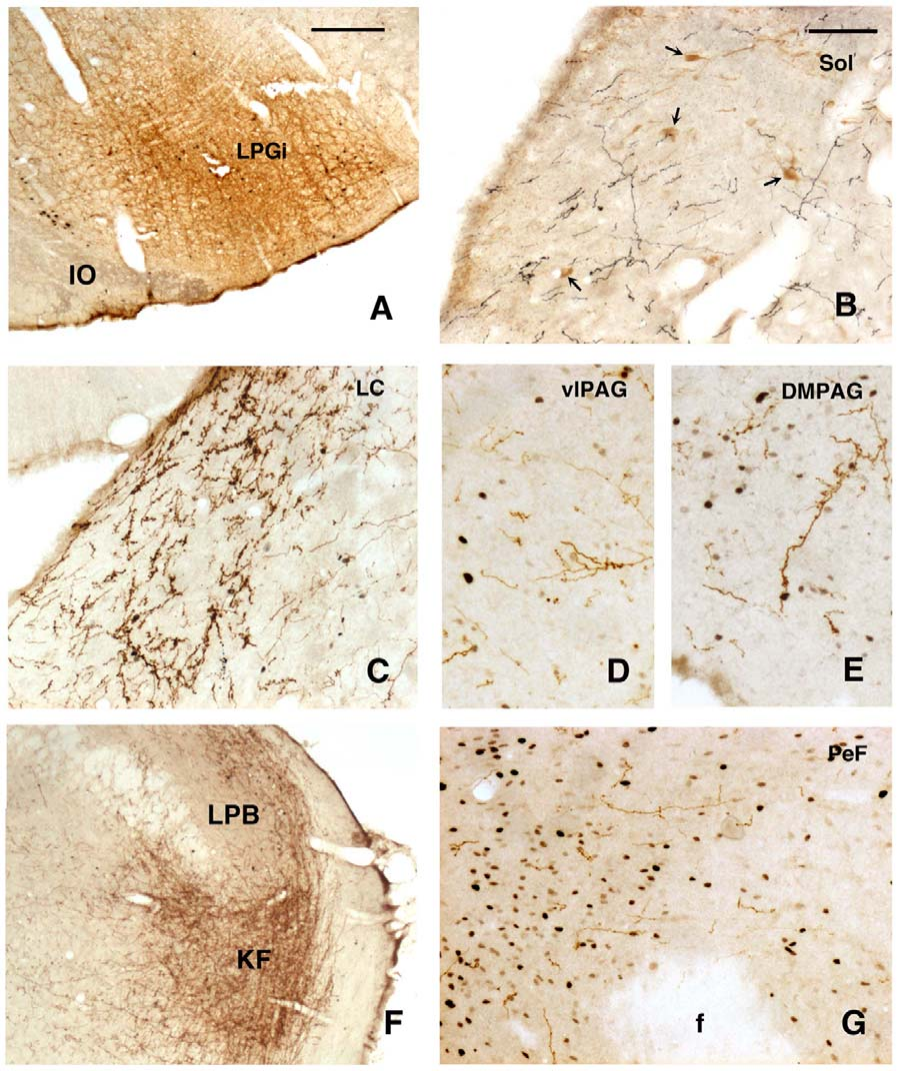
Anterograde tracing with PHA-L visualized after injection in the LPGi . A. Injection site. B–G. Anterogradely labelled axons are observed among the catecholaminergic neurons (arrows) of the nucleus of the solitary tract (Sol) (B), in the locus coeruleus (LC) (C), in the ventrolateral periaqueductal gray (vlPAG) (D) and dorsomedial periaqueductal gray (DMPAG) (E), in the Kölliker-Fuse (KF) and lateral parabrachial nuclei (LPB) (F) and in the perifornical area of the hypothalamus (PeF) (G). Fos-immunoreactive nuclei, induced by the PS-recovery condition are visible in D, E and G. Abbreviations: f, fornix, IO, inferior olive. Bar  = 500 µm in A and F, 75 µm in B-E and G.

These projections were determined with injections of PHA-L. As mentioned before for the electrophysiological recording, only the sites located in the main part of the LPGi (Fig. 4A), caudally to the facial nucleus, were considered. Upon examination of the labeling it was observed that the LPGi had extensive axonal projections. In every region, non-varicose axons were mixed with varicose axons, sometimes emitting convoluted collaterals bearing terminal-like boutons. However, the non-varicose axons were more numerous in the brainstem, close to their source.

Similarly to the description by Guyenet and Young [18], the majority of the axons emanating from the LPGi coursed in the ipsilateral side of the brain but the ipsi- vs the contralateral difference was attenuated at rostral levels. At medullary level, an important contingent of axons was observed to cross the midline and innervate the contralateral LPGi. From there, some axons ascended obliquely in the medullary reticular formation, symmetrically to a more important ipsilateral group of axons, and reached the hypoglossal nucleus and the solitary tract nucleus, (Fig. 4B). Just rostral to the LPGi, the facial nucleus was populated by a significant number of axons coursing among its motoneurons (see below). The axons continued their course laterally through the noradrenergic A5 group and expanded over the superior olive in the ventral pontine reticular formation. The core of the medullary and pontine reticular formation was populated with a low number of axons. From the A5 group, the axons ascended medially and laterally to the motor trigeminal nucleus. They innervated the LC with a substantial density (Fig 4C) and the Kölliker-Fuse and lateral parabrachial nuclei with a high density (Figs. 4F). The rostral continuation of this pathway reached the periaqueductal gray (PAG) and was distributed over all its subdivisions with conspicuous groups of axons in the ventrolateral (Fig. 4D), lateral and dorsomedial (Fig. 4E) subdivisions. Very few axons were observed in the mesencephalic reticular formation and only isolated axons in the ventral tegmental area. Several subdivisions of the hypothalamus contained a loose network of axons, namely the perifornical (Fig. 4G) and lateral hypothalamic areas, the dorsomedial nucleus and the zona incerta. The paraventricular nucleus displayed a very small number of axons in its parvicellular subdivision. Axons were present rostrally to the hypothalamus, but they were always isolated, in the median and lateral preoptic areas, the bed nucleus of the stria terminalis, and the accumbens nucleus. Some of them reached the deep layers of the prefrontal cortex and travelled radially over all layers of the motor cortex.

**Figure 5:**
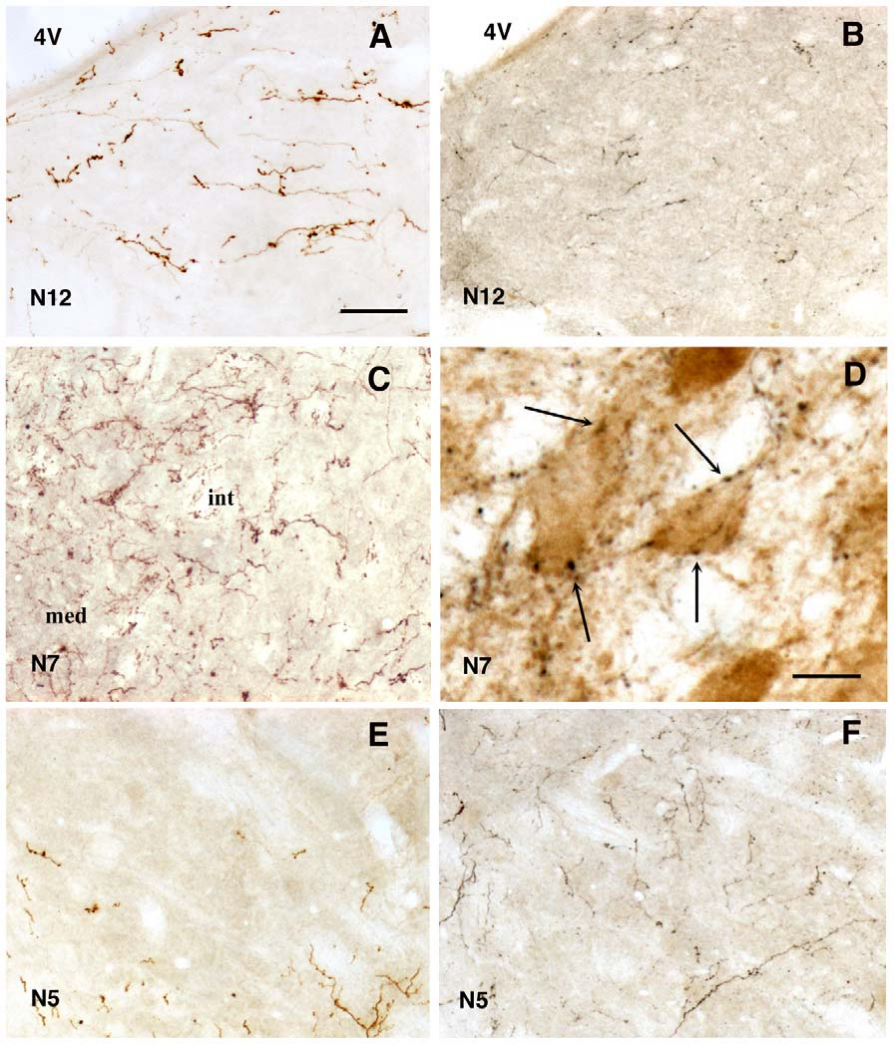
Anterogradely labelled axons in the cranial motor nuclei after injection of PHA-L in the LPGi. Axons in the hypoglossal (N12) (A, B), facial (N7) (C, D) and trigeminal (N5) (E, F) motor nuclei after injection of PHA-L in the caudal part (A, C, E) and rostral part (B, D, F) of the LPGi. In D note the labelled varicosities (arrows) adjacent to the choline acetyltransferase-immunoreactive motoneurons in the medial part of the nucleus. Abbreviations: 4V, fourth ventricle, int, and med, intermediate and medial parts of the facial nucleus. Bar  = 70 µm in A,B,C,E and F, 30 µm in D.

Deserving emphasis is the topographical origin of the innervation of the cranial motor nuclei by the LPGi. When the PHA-L injection covered the caudal third of the LPGi, numerous axons invaded the hypoglossal nucleus (Fig. 5A), whereas few or very few axons impinged on the trigeminal nucleus (Fig. 5E). The opposite was true when the injection of PHA-L was located in the part of the LPGi just caudal to the facial nucleus (Fig. 5B, F). In the facial nucleus, PHA-L axons were seen after both types of injection. In this nucleus, the axons ran among the motoneurons of all subdivisions (Fig. 5C) and often covered the periphery of the laterodorsal subdivision. Within the nucleus, many of their varicosities were closely apposed to the cell bodies of motoneurons (Fig. 5D).

#### Afferent connections of the facial nucleus activated during PS hypersomnia

**Figure 6:**
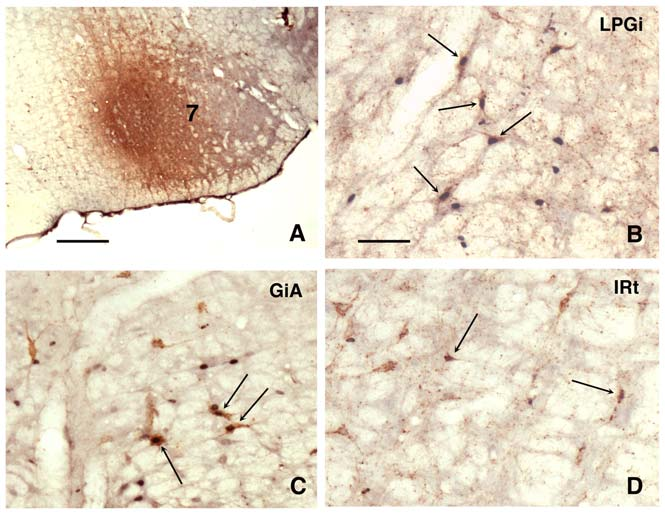
Fos/CTb labeling in the PS recovery condition after injection of CTb in the facial nucleus. A: CTb injection site. Double-labelled neurons (arrows) in the ipsilateral LPGi (B), in the ipsilateral gigantocellular reticular nucleus, alpha part (GiA)(C) and in the contralateral intermediate reticular nucleus (IRt) (D). Bar  = 500 µm in A, 60 µm in B–D.

Given the absence of muscle tone in the muscles of the face during PS [33], [34], our attention was drawn by the presence, in the facial nucleus, of axons issued from the LPGi, whatever the location of the injection site. In view of this observation, we decided to explore the possibility that the LPGi might participate in the silencing of the facial motoneurons during PS through activation of its axonal projection to this nucleus. This is all the more interesting in view of the paucity of data regarding the anatomical origin of the axons underlying the hyperpolarization of the facial motoneurons [35]. To this aim, we injected CTb in the facial nucleus (Fig. 6A) in 3 rats, using the same protocol as for the injections in the LPGi. The rats were then submitted to the PS deprivation protocol and perfused after 3h of PS recovery. The single CTb- and double-labelled Fos/CTb neurons were counted on 5 sections taken at 600- µm intervals across the medullary levels containing the LPGi and the facial nucleus (Bregma -13.30, -12.70, -12.10, -11.50 and -10.90 mm) (see Materials and Methods).

**Figure 7:**
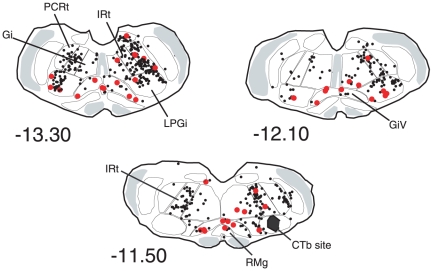
Distribution of the single CTb (black dots) and double-labelled Fos/CTb (red dots) in a PS-recovery rat after an injection of CTb in the facial nucleus (Blackened area at -11.50). Figures refer to the distance from the Bregma. Abbreviations : Gi, gigantocellular reticular nucleus; GiV, gigantocellular reticular nucleus, ventral part; IRt, intermediate reticular nucleus; PCRt, parvicellular reticular nucleus.

The distribution of the CTb labelled neurons was in full agreement with the data of other authors [36], [37], [38]. The majority of the retrogradely labelled neurons were located in the neighbouring reticular formation, i.e. gigantocellular, parvocellular and intermediate reticular nuclei (Figs 6, 7 and Table 2). The intermediate reticular nucleus displayed the highest number of single CTb neurons. All the afferents were predominantly ipsilateral (62% of the retrogradely-labelled neurons were counted ipsilaterally to the injected facial nucleus). The highest number of double-labelled Fos/CTb neurons was counted in the LPGi (Table 2), which represented 28% of the double-labelled neurons counted in the medulla. The second more important percentage (20%) was in the gigantocellular reticular nucleus, ventral part (GiV). The intermediate reticular nucleus contained 16% of the double-labelled neurons.

**Table 2:** Number (mean ± SEM) of single-CTb and double Fos/CTb neurons after CTb injection in the facial nucleus. Counts were made in 3 rats during PS recovery following a 72h PS-deprivation.

	Nb of sections	contralateral CTb	ipsilateral CTb	contralateral Fos/CTb	ipsilateral Fos/CTb	%Fos/CTb vs total Fos/CTb
GiA	2	8,33±1,20	12,67±1,67	2,67±1,16	3±1,36	15,04
Gi	5	30±6,00	47±9,01	1,67±0,81	2±1,28	9,73
GiV	3	8±1,53	12±2,65	4±1,00	3,67±0,33	20,35
Irt	5	86,33±7,22	109±15,58	2,33±1,42	3,67±0,81	15,93
LPGi	5	42±14,01	66,33±27,75	3,67±0,87	7±0,87	28,32
PCRt	5	39,33±8,03	106±0,58	0,33±0,29	2,67±0,58	7,96
RMg	2	1±0,00		5±1,16		2,65

Abbreviations: Gi : gigantocellular reticular nucleus; GiA : gigantocellular reticular nucleus, alpha part; GiV : gigantocellular reticular nucleus, ventral part; Irt : intermediate reticular nucleus; LPGi : lateral paragigantocellular nucleus; PCRt : parvocellular reticular nucleus; RMg : raphe magnus nucleus.

#### Afferent connections of the LPGi activated during PS deprivation and recovery

**Figure 8:**
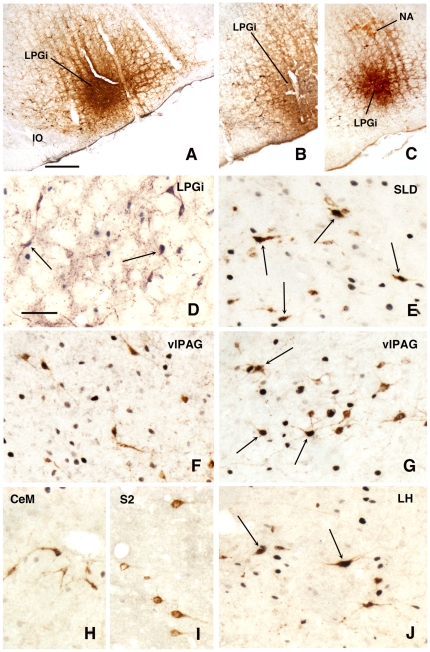
Fos (or TH or ChAT)/CTb labeling after injection of CTb in the LPGi. A–F and –HJ: PS-recovery condition, G: PS-deprived condition. A: CTb injection site in the LPGi covering Fos nuclei, B: CTb injection site in the LPGi covering TH- neurons of the A1/C1 group, C: CTb injection site in the LPGi, ventrally to the ChAT- neurons of the ambiguus nucleus (NA), D: Contralateral LPGi, E: sublaterodorsal nucleus (SLD) showing many double-labelled Fos-CTb neurons, F: ventrolateral periaqueductal gray (vlPAG) in the PS-recovery condition, G: ventrolateral periaqueductal gray (vlPAG) in the PS-deprivation condition, H: single CTb neurons in the central nucleus of the amygdala (CeM), I: single CTb neurons in the pyramidal layer of the secondary somatosensory cortex (S2), J: lateral hypothalamus (LH). Arrows indicate double-labelled Fos-CTb neurons. Bar  = 500 µm in A-C and 60 µm in D-J.

In order to understand the role of the LPGi during PS, the following step was to localize the neurons controlling its activity during PS. This was accomplished by injecting CTb in the LPGi of 12 animals: 4 control rats (PSC), 4 rats deprived of PS (PSD) and 4 rats recovering PS (PSR). The retrogradely labelled neurons expressing Fos were plotted in all the animals (see Materials and Methods). Similarly to the PHA-L injections, only the sites located in the main part of the LPGi (Fig. 8A,B,C) were considered. They were in the area of the catecholaminergic neurons of the A1/C1 group (Fig. 8B) and ventral to the cholinergic neurons of the ambiguus nucleus (Fig. 8C).

**Figure 9:**
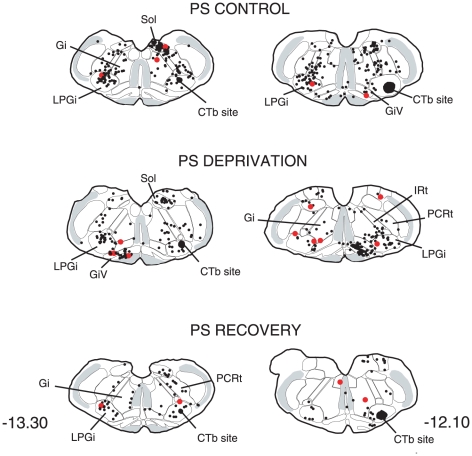
Distribution of the single CTb (black dots) and double-labelled Fos/CTb (red dots) neurons, as observed in the medulla of one PS control, one PS-deprived and one PS-recovery rat after injection of CTb in the LPGi, in the sides ipsilateral (right) and contralateral (left) to the injection site (blackened area). Figures refer to the distance from the Bregma. Abbreviations : Gi: gigantocellular reticular nucleus; GiV: gigantocellular reticular nucleus, ventral part; IRt: intermediate reticular nucleus; LPGi: lateral paragigantocellular nucleus; PCRt: parvicellular reticular nucleus; Sol: nucleus of the solitary tract.

**Figure 10:**
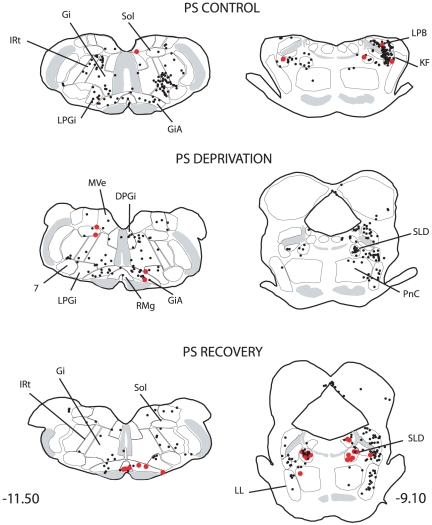
Distribution of the single CTb (black dots) and double-labelled Fos/CTb (red dots) neurons, as observed in the medulla and pons of one PS control, one PS-deprived and one PS-recovery rat after injection of CTb in the LPGi, in the sides ipsilateral (right) and contralateral (left) to the injection site. Figures refer to the distance from the Bregma. Abbreviations: 7: facial nucleus; DPGi: dorsal paragigantocellular nucleus; Gi: gigantocellular reticular nucleus; GiA: gigantocellular reticular nucleus, alpha part; IRt: intermediate reticular nucleus; KF: Kölliker-Fuse nucleus; LL: ventral nucleus of the lateral lemniscus; LPB: lateral parabrachial nucleus; LPGi: lateral paragigantocellular nucleus; PCRt: parvicellular reticular nucleus; MVe: medial vestibular nucleus; PnC: pontine reticular nucleus, caudal part; RMg: nucleus raphe magnus; SLD: sublaterodorsal nucleus; Sol: nucleus of the solitary tract.

**Figure 11:**
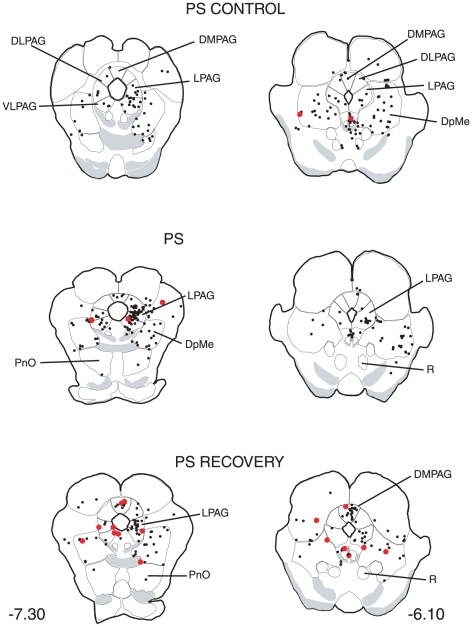
Distribution of the single CTb (black dots) and double-labelled Fos/CTb (red dots) neurons, as observed in the pons and mesencephalon of one PS control, one PS-deprived and one PS-recovery rat after injection of CTb in the LPGi, in the sides ipsilateral (right) and contralateral (left) to the injection site. Figures refer to the distance from the Bregma. Abbreviations: DMPAG: dorsomedial periaqueductal gray; DLPAG: dorsolateral periaqueductal gray; DpMe: deep mesencephalic nucleus; LPAG: lateral periaqueductal gray; PnO: pontine reticular nucleus, oral part; R: red nucleus; VLPAG: ventrolateral periaqueductal gray.

**Figure 12:**
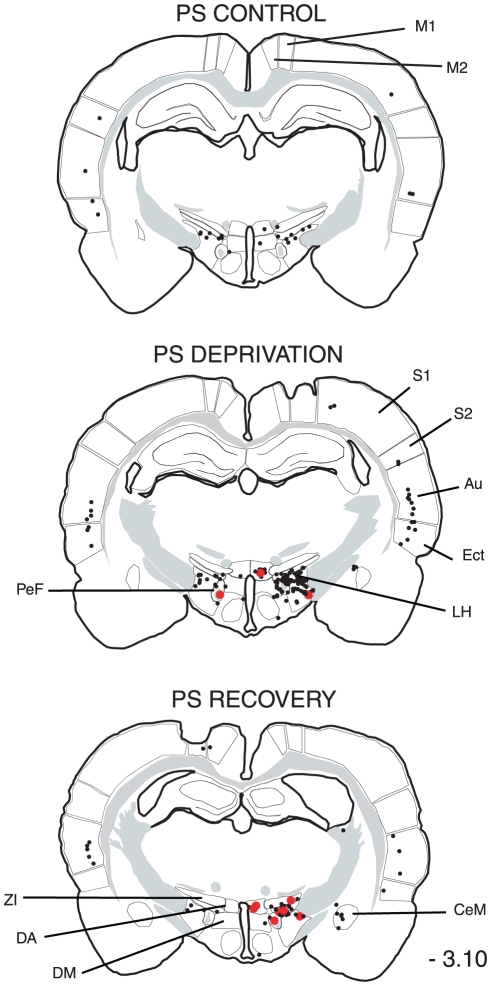
Distribution of the single CTb (black dots) and double-labelled Fos/CTb (red dots) neurons, as observed in the hypothalamus and cortex of one PS control, one PS-deprived and one PS-recovery rat after injection of CTb in the LPGi, in the sides ipsilateral (right) and contralateral (left) to the injection site. Figures refer to the distance from the Bregma. Abbreviations: Au: auditory cortex; CeM: central amygdaloid nucleus; DA: dorsal hypothalamic area; DM: dorsomedial hypothalamic nucleus; Ect: ectorhinal cortex; LH: lateral hypothalamic area; M1: primary motor cortex; M2: secondary motor cortex; PeF: perifornical nucleus; S1: primary somatosensory cortex; S2: secondary somatosensory cortex; ZI: zona incerta.

**Figure 13:**
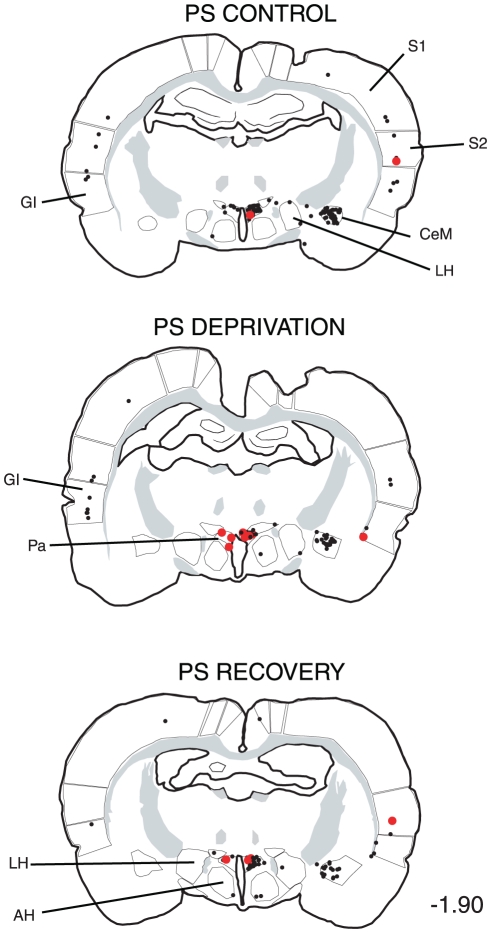
Distribution of the single CTb (black dots) and double-labelled Fos/CTb (red dots) neurons, as observed in the rostral hypothalamus and cortex of one PS control, one PS-deprived and one PS-recovery rat after injection of CTb in the LPGi, in the sides ipsilateral (right) and contralateral (left) to the injection site. Figures refer to the distance from the Bregma. Abbreviations: AH: anterior hypothalamic area; CeM: central amygdaloid nucleus; GI: insular cortex; LH: lateral hypothalamic area; Pa: paraventricular hypothalamic nucleus; S1: primary somatosensory cortex; S2: secondary somatosensory cortex.

**Figure 14:**
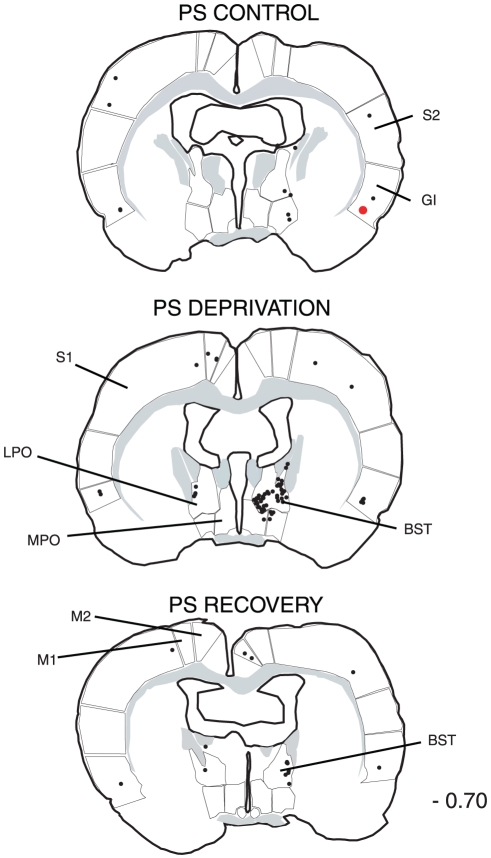
Distribution of the single CTb (black dots) and double-labelled Fos/CTb (red dots) neurons, as observed in the basal forebrain and cortex of one PS control, one PS-deprived and one PS-recovery rat after injection of CTb in the LPGi, in the sides ipsilateral (right) and contralateral (left) to the injection site. Figures refer to the distance from the Bregma. Abbreviations: BST: bed nucleus of the stria terminalis; GI: insular cortex; LPO: lateral preoptic area; M1: primary motor cortex; M2: secondary motor cortex; MPO: medial preoptic area; S1: primary somatosensory cortex; S2: secondary somatosensory cortex.

**Figure 15:**
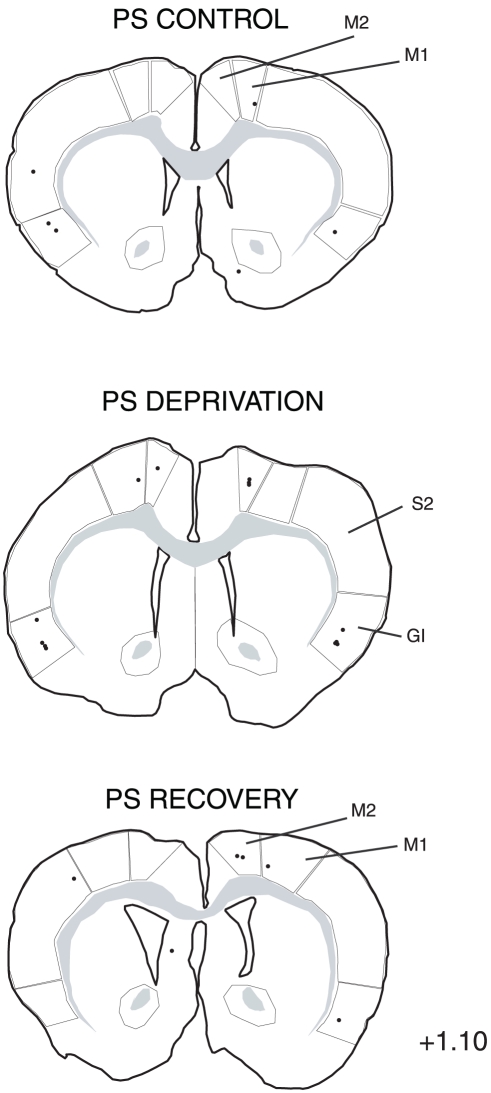
Distribution of the single CTb (black dots) and double-labelled Fos/CTb (red dots) neurons, as observed in the cortex of one PS control, one PS-deprived and one PS-recovery rat after injection of CTb in the LPGi, in the sides ipsilateral (right) and contralateral (left) to the injection site. Figures refer to the distance from the Bregma. Abbreviations: GI: insular cortex; M1: primary motor cortex; M2: secondary motor cortex; S1: primary somatosensory cortex; S2: secondary somatosensory cortex.

The distribution of the single CTb and double Fos/CTb neurons is illustrated in Figs. 9, 10, 11, 12, 13, 14, 15 for the 10 most representative frontal planes. The localization of the CTb neurons was in very close correspondence with previous studies devoted at determining the afferents of the LPGi [21], [22], [23], [24], [39], [40], [41], [42], [43]. A few additional neurons were disclosed here in the auditory and motor neocortex.

The number of neurons in these two categories was quantified in 40 nuclei corresponding to areas where retrogradely labelled neurons were constantly observed. The numbers counted in each area and each experimental condition are displayed in Table 3. When the sum of the ipsi- and contralateral sides is made, it appears that the largest number of single CTb- neurons was counted in the LPGi (117 neurons) (Table 3, Figs. 9–10). In decreasing order after the LPGi in the brainstem, were the deep mesencephalic nucleus (DpMe) (105 neurons) (Fig. 11), the lateral periaqueductal gray (LPAG) (93 neurons) (Fig. 11), the gigantocellular reticular nucleus (79 neurons) (Figs. 9–10), the ventrolateral PAG (vlPAG) (Figs. 8F,G, 11) and the solitary tract nucleus (70 neurons each) (Figs. 9–10). Rostrally, a substantial number of retrogradely labelled neurons were observed in the lateral hypothalamic area (68 neurons) (Figs. 8J, 12–13), the central nucleus of the amygadala (39 neurons) (Figs. 8H, 12–13) and two cortical areas, namely the somatosensory (28 neurons) (Figs. 8I, 12, 13, 14, 15) and the insular cortex (41 neurons) (Figs. 13, 14, 15).

**Table 3:** Numbers (mean ± SEM) of single CTb and double-labelled Fos/CTb neurons in 40 brain areas after injection of CTb in the LPGi in the PS-control (PSC), PS-deprived (PSD) and PS-recovery (PSR) conditions.

				PSC		PSD		PSR	
	*n*	CTb contra	CTb ipsi	Fos/CTb contra	Fos/CTb ipsi	Fos/CTb contra	Fos/CTb ipsi	Fos/CTb contra	Fos/CTb
									ipsi
**Medulla oblongata**									
LPGi	5	69,5±14,20	48,42±4,50	1±0,41	0,25±0,25	2,25±0,85	3±0,57	3,5±2,25	2±1,41
DPGi	3	6,75±1,69	2,83±0,47	0	0	0,25±0,25	0,25±0,25	1,75±1,03	0,75±0,48
Gi	6	34,67±7,22	44,17±9,17	0,25±0,25	0,25±0,25	0,5±0,29	1,75±0,75	1±0,57	2,25±1,31
Irt	6	20,92±4,19	23,67±4,97	0	0	0,5±0,29	0,25±0,25	0,5±0,50	0,75±0,48
PCRt	6	23,58±5,39	37,58±9,57	0,75±0,48	0,25±0,25	0,5±0,29	1±0,57	0	1±0,70
GiV	3	9,92±2,51	8,92±0,92	0	0,25±0,25	0,75±0,48	0	1±1,00	0,75±0,48
GiA	3	13,08±2,11	26,58±5,85	0	0	0,25±0,25	4,5±2,84	2,25±0,94	4,5±2,10^***^
Sol	4	11±0,69	59,09±15,09	0	0,25±0,25	0,25±0,25	1,75±1,03	0,5±0,29	1,5±1,50
Rob	4	9,42±1,49		0		0		1±0,70	
Rpa	5	5,33±0,61		0		0,25±0,25		0,25±0,25	
RMg	4	13±2,56		0		1,5±0,64		2,25±0,48^***^	
Mve	5	5,42±1,91	7,42±2,55	0,25±0,25	0,00	0,00	0,5±0,50	0,00	0,25±0,25
**Pons**									
KF	1	2,58±0,07	13,42±2,34	0,5±0,29	0,5±0,29	0	0,25±0,25	0,25±0,25	0,25±0,25
A5	2	3,83±1,47	9,83±3,66	0,00	0,00	0,25±0,25	0,25±0,25	0,00	0,5±0,29
LPB	2	4,42±0,26	32,5±8,43	0	1,5±0,95	0,25±0,25	0,75±0,48	0,25±0,25	0,75±0,48
MPB	2	3,25±0,69	10,83±1,87	0	0	0	1,25±0,94	0	0,5±0,29
PnV	1	1,92±0,26	4,17±1,42	0	0	0	0	0	0,5±0,29
PnC	2	12±1,73	14,17±2,88	0,25±0,25	0	1,25±0,48	0	0,75±0,25	0,25±0,25
PnO	3	9,42±1,46	14±3,46	0,25±0,25	0	1±0,70	0,75±0,75	0,25±0,25	1,25±0,63
SLD	2	8,5±1,65	12,33±0,95	0,25±0,25	1,75±1,18	0,25±0,25	0	4,75±2,49	6,75±2,25**^###^**
**Mesencephalon**									
DpMe	5	42,62±6,64	62,58±8,07	0,25±0,25	0,5±0,29	0	0	2,75±1,37	4,5±1,32^*,###^
DMPAG	6	27,58±4,82		0,25±0,25		0,5±0,50		3,25±0,63^***,#^	
DLPAG	6	5,83±0,97	13,75±3,15	0	0	0,5±0,29	0,25±0,25	0,5±0,50	1,5±0,64
LPAG	6	26,33±4,40	67±14,57	0,5±0,29	0,25±0,25	0,5±0,50	3±0,70^***^	2±0,41	4,5±1,44^*,#^
vlPAG	3	17,75±5,41	53,83±13,23	0,25±0,25	0,5±0,29	1,25±0,94	2,5±0,29^***^	2,75±0,63	4,25±2,62
**Hypothalamus**									
antPAG	2	6,33±1,33	9,42±1,22	0	0,25±0,25	0	0,75±0,48	0	1±0,00
PH	3	4,25±0,50	7,58±0,07	0	0,25±0,25	0	0	0	0
DA	2	4,25±0,50	9,17±1,89	0	0,25±0,25	0	0,5±0,29	0,5±0,29	1±0,41
DM	2	2,33±0,52	3,42±1,65	0	0	0,25±0,25	0,25±0,25	0	0,5±0,29
LH	6	16,33±4,99	52,17±13,80	0	1±0,57	1,5±1,19	3,5±1,32	1±0,41	6,25±1,37^***^
Pa	1	6,17±1,13	34,33±6,24	0	0,5±0,29	0,75±0,75	3,5±2,02	0,25±0,25	0,75±0,48
PeF	1	1,67±0,38	3,92±1,28	0	0	0,75±0,48	0,5±0,29	0,5±0,50	1,25±0,48
ZI	5	4,50±0,33	15,75±3,75	0	0,25±0,25	0	0,25±0,25	0	1,5±0,95
**Amygdala**									
CeM	2	0,92±0,50	38,25±6,71	0	0	0	0,25±0,25	0	0,75±0,48
**BST**	2	1,5±0,45	12,75±6,10	0,25±0,25	0,00	0,00	0,00	0,00	0,00
**Cortex**									
Ect	2	3,42±1,28	4,08±1,13	0,00	0,00	0,00	0,00	0,00	0,25±0,25
Au	2	6,50±2,62	9,17±3,26	0,00	0,00	0,00	1,25±0,75	0,00	0,5±0,29
GI	7	18,00±5,20	23,42±3,88	0,00	0,25±0,25	0,75±0,48	0,5±0,29	0,00	0,00
S1	9	13,58±2,65	14,92±4,07	0,00	0,25±0,25	0,00	0,5±0,29	0,00	0,5±0,50
M1	8	4,67±2,19	5,00±1,68	0,00	0,00	0,25±0,25	0,00	0,00	0,00

Legend of Table 3. The values displayed are an average, across 4 animals in each condition, of the sum of the neurons counted at 600 µm intervals through the full extent of each nucleus. hello P≤0.05 ; hellohellohello P≤0.001 vs PSC ; # P≤0.05 ; ### P≤0.001 vs PSD. Abbreviations: A5: A5 noradrenergic area; antPAG: anterior periaqueductal gray; Au: auditory cortex; BST: bed nucleus of the stria terminalis; CeM: central amygdaloid nucleus; DA: dorsal hypothalamic area; DM: dorsomedial hypothalamic nucleus; DMPAG: dorsomedial periaqueductal gray; DLPAG: dorsolateral periaqueductal gray; DPGi: dorsal paragigantocellular nucleus; DpMe: deep mesencephalic nucleus; Ect: ectorhinal cortex; Gi: gigantocellular reticular nucleus; GI: insular cortex; GiA: gigantocellular reticular nucleus, alpha part; GiV: gigantocellular reticular nucleus, ventral part; Irt: intermediate reticular nucleus; KF: Kölliker-Fuse nucleus; LH: lateral hypothalamic area; LPAG: lateral periaqueductal gray; LPB: lateral parabrachial nucleus; LPGi: lateral paragigantocellular nucleus; M1: primary motor cortex; MPB: medial parabrachial nucleus; MVe: medial vestibular nucleus; Pa: paraventricular hypothalamic nucleus; PCRt: parvicellular reticular nucleus; PeF: perifornical nucleus; PH: posterior hypothalamic nucleus; PnC: pontine reticular nucleus, caudal part; PnO: pontine reticular nucleus, oral part; PnV: pontine reticular nucleus, ventral part; RMg: raphe magnus nucleus; RPa: raphe pallidus nucleus; ROb: raphe obscurus nucleus; S1: primary somatosensory cortex; SLD: sublaterodorsal nucleus; Sol: nucleus of the solitary tract; vlPAG: ventrolateral periaqueductal gray; ZI: zona incerta.

Apart from the LPGi and the dorsal paragigantocellular nucleus, the number of CTb neurons was highest ipsilaterally. The nucleus that had the highest ipsilateral versus contralateral contribution was the central nucleus of the amygdala (38 neurons vs 1 neuron). On the contrary, all the medullary and pontine reticular nuclei showed a very modest ipsilateral dominance. This was also the case of all the cortical areas. Overall, the contribution from the ipsilateral side was 65% of the sum of both sides.

As concerns the double-labelled Fos/CTb neurons, they also showed a predominant ipsilateral distribution. In the control condition, their number was very low in all nuclei (from 2 neurons to less than one neuron, as an average) (Table 3). In the PS-deprived condition, it was slightly higher in most nuclei. In this PSD condition, the only areas where the number was significantly different from control were the LPAG and vlPAG (Figs. 8G, 11) (3 neurons versus less than one neuron as an average, p<0,001)). In the PS-recovery condition, the number was higher than PSC (Table 3) in the gigantocellular reticular nucleus, alpha part (GiA) (p<0.001), the raphe magnus nucleus (p<0.001), the DpMe (p<0,05), the dorsomedial PAG (DMPAG) (p<0.001) (Fig. 11) and the lateral hypothalamic area (p<0.001) (Figs. 8J, 12–13). They were higher than PS-deprived (Table 3) in the SLD (p<0.001) (Figs. 8E, 10), the DpMe (p<0.001), the DMPAG (p<0.05) and the LPAG (p<0.05). It is noticeable that in the PS-recovery condition, the SLD showed the greatest number of Fos/CTb labelled neurons among all nuclei showing double-labelled neurons (7 neurons ipsilaterally and 5 contralaterally), which represented 55% of the CTb-labelled neurons in the ipsilateral SLD and 56% in the contralateral SLD.

## Discussion

### Electrical activity of the LPGi neurons during the sleep-waking cycle

As developed several years ago for the rat [28], the head-restraining system is a semi-chronic preparation allowing a combination of single-unit and polygraphic recordings under painless and anesthetic-free stereotaxic conditions. Like previous authors taking advantage of the same preparation [15], [44] we observed, along the course of all recording sessions, a sleep-waking cycle organization similar to that of freely moving rats.

The first salient data of this work is that it provides the first electrophysiological recordings of LPGi neurons during the sleep-waking cycle in the rat. These data validate our previous observations for the presence of neurons expressing Fos in the PS-deprivation and PS-recovery conditions [12], [13], [14]. The present results show that the LPGi contains neurons selectively active during PS (PS-On) neurons, but also neurons selectively inactive during this state (PS-Off neurons) and neurons the discharge of which is not related to the sleep-waking cycle. Thus, the LPGi appears to be a heterogeneous structure with regard to the regulation of the sleep-waking cycle.

The neurons considered here as PS-On neurons satisfy the criteria of PS-On neurons, as defined by Sakai [17], [45] for the cat brainstem, by showing i) a low or very low rate of discharge during waking, ii) a significant increase in discharge rate a few seconds prior to the onset of PS and iii) a tonic mode of discharge during PS. Neurons with a similar electrical profile across the sleep–waking cycle were recorded in rat and cat species, in different nuclei of the brainstem and hypothalamus. Interestingly, they were found in the area equivalent to the LPGi in the cat ventrolateral medulla [17], [45]. They were also found in the pontine area regarded as the executive centre of PS (SLD in the rat and peri-LC alpha in the cat) [17], [45], [46], [47], in the dorsal paragigantocellular nucleus [48] and in the caudal and lateral parts of the hypothalamus [26], [27], [49], [50], [51], [52]. These findings have greatly extended our knowledge of the network regulating the generation of PS and prompt us to suggest that the PS-On neurons recorded here in the LPGi are an integral part of this network.

The neurotransmitter expressed by these PS-On neurons is so far unknown. From the works by Boissard et al [44] and Sapin et al [14], it is however likely that most if not all of them are glycinergic and/or GABAergic. Indeed, about one-third of the neurons expressing Fos in the LPGi during a PS hypersomnia also express glycine [44] and 70% express GAD, the synthetic enzyme of GABA [14]. Besides, from the anatomical study by Stornetta et al [53], it is known that, in this part of the brain, many neurons express both neurotransmitters. The hypothesis of the GABAergic nature of some PS-On neurons is supported by electrophysiological recordings establishing that GABAergic neurons in different areas of the brain, e.g. the dorsal raphe nucleus [54], the amygdala [55] and the neocortex [56] emit spikes of short duration, around 1 ms, like the PS-On neurons in the LPGi. Implementing juxtacellular labelling followed by immunohistochemical identification of the vesicular transporter for GABA in their axons (see [27] might be a way to confirm the GABAergic nature of the PS-On neurons.

The PS-Off neurons discharge in a reciprocal manner to the PS-On neurons. As suggested for other regions of the brain [27], it is possible that both populations reciprocally inhibit each other in the LPGi by progressively increasing their tonus on the other population. This is concordant with the fact that the PS-Off neurons start to decrease their firing slightly before the onset of PS and the PS-On neurons increase their firing several seconds before the PS episodes. Similarly to the PS-On neurons, part of the PS-Off neurons could be GABAergic. Indeed, we have shown that in the experimental condition requiring the inhibition of PS, i.e. during the 72h-PS deprivation, 55% of the neurons expressing Fos are GABAergic in nature [14]. In concordance with the mutual inhibition hypothesis, it has been suggested that the numerous GABAergic neurons in the ventrolateral medulla might act in part as locally projecting neurons [57]. However, an adrenergic nature of some of the PS-Off neurones is also possible. Among the adrenergic neurons belonging to the C1 group spread over the LPGi, one-fifth express Fos at the end of a 72h-PS deprivation [16]. Moreover, the low discharge rate of the PS-Off neurons during W and SWS (4–5 Hz on the average) is included in the range of the discharge rate of C1 adrenergic neurons (1–10 Hz), as recorded in the anaesthetized rat [58], [59], [60]. It is also similar to the rate of the LC noradrenergic neurons as identified in the head-restrained preparation [15].

### Presumed roles of the PS-On LPGi neurons during the sleep-waking cycle

#### Inhibition of distant and local PS-Off neurons

It has been known for 3 decades that the noradrenergic LC neurons and most of the serotonergic neurons in the dorsal raphe nucleus cease firing during PS, i.e. show a PS-Off or wake-promoting activity [61], [62], [63]. Using pharmacology in the head-restrained rat and the combination of anatomy and PS deprivation-recovery in previous studies, we have demonstrated that this inhibitory drive is of GABAergic nature and suggested several possible sources for it [13], [14], [15], [48], [64], [65]. One of these sources is the LPGi since it contains i) a substantial number of neurons active during PS and projecting to the LC [13] and ii) neurons expressing Fos and GAD during PS hypersomnia [14]. In support of a monosynaptic inhibitory projection, Ennis & Aston-Jones [66] have shown that, in addition to an excitatory effect, the stimulation of the LPGi elicits a clear inhibitory response with a short latency in a set of LC neurons. Interestingly, the LPGi has the ability to simultaneously influence the LC and the dorsal raphe nucleus because some of its neurons send axonal collaterals to both nuclei [67].

These nuclei are not the only ones through which the LPGi might control the sleep-waking cycle. Good candidates are represented by different subdivisions of the PAG. Indeed, the vlPAG receives a substantial number of axons from the LPGi [22], [68] (present work) and contains GABAergic PS-On and PS-Off neurons identified as critical elements for the generation of PS [3], [8], [14], [69]. The inactivation of these PS-Off neurons, a required step for PS onset, is induced pharmacologically by local injection of muscimol, a GABA_A_ agonist [14], [69] and, during the physiological sleep-wake cycle, could originate in part from PS-On neurons in the LPGi. Given the relatively modest number of LPGi axons observed here rostrally to the PAG, e.g. in the hypothalamus where the PS-Off orexin/hypocretin neurons are located, it is tempting to speculate that the control exerted by the LPGi PS-On neurons over PS-Off neurons is limited to the brainstem, in the aminergic nuclei and the vlPAG. As suggested above, the PS-On neurons might also inhibit the local PS-Off neurons.

#### Control of the facial nucleus during PS

The second major findings of this study is that a set of the LPGi neurons sending axons onto the facial nucleus is activated during PS hypersomnia. With nearly 30% of the double-labelled neurons, the LPGi contains the highest percentage of activated neurons among the medullary nuclei. The parvocellular and intermediate reticular nuclei, known as the major sources of medullary afferents to the facial nucleus [36], [37], [38] display lower percentages, 8 and 16%, respectively. These results emphasize the potential role of the LPGi in the hyperpolarization of the facial motoneurons during PS. As largely documented for the trigeminal, hypoglossal and spinal motoneurons, this hyperpolarization has long been thought to be solely due to glycinergic inputs [70], [71], [72] but recent data suggest that, in addition to glycine, GABAergic neurons would be involved [73]. As illustrated in anatomical works, it has been postulated that the cell bodies of these glycinergic and GABAergic neurons belong to three common pools of neurons located i) around the trigeminal motor nucleus, ii) in the ventromedial medulla (GiA and GiV) and iii) in the parvocellular and intermediate reticular nuclei [74], [75], [76]. This holds true for the facial nucleus as well [75]. However, in the study by Morales et al [77], aimed at localizing in the cat the neurons activated during PS (induced by carbachol administration) and projecting to the trigeminal nucleus, such cell bodies were exclusively found in a small area encompassing the lateral part of the GiV and the rostral LPGi. In the present work we found, exactly in the same area, neurons projecting to the facial nucleus. We also found neurons in the ventromedial medulla (GiA and rest of the GiV) and the core of the LPGi. Our results suggest for the first time that the LPGi GABAergic and/or glycinergic PS-On neurons play a major role in the hyperpolarization of the facial motoneurons during PS. Further, they suggest that this hyperpolarization arises from GABA and glycinergic neurons localized in three different medullary areas, contrary to previous hypotheses of a restricted localization in the GiV and GiA. Therefore, it is conceivable that the neurons of these three areas form an anatomical and physiological continuum, those in the LPGi being directed towards the cranial motoneurons and those in the GiV and GiA being mainly aimed at the spinal motoneurons.

### Neurons regulating the activity of the PS-On and PS-Off neurons in the LPGi

The third most salient result of our study is that the SLD, known to contain the neurons triggering PS [3], [44], hosts the highest number of Fos/CTb neurons among the areas showing double-labelled neurons in the PSR condition after CTb injection in the LPGi. Interestingly, in this nucleus the double-labelled neurons represent 50% of the retrogradely labelled neurons, which is by far the highest percentage among nuclei bearing double-stained neurons. Further, they are nearly equally distributed between the ipsi- and the contralateral sides. It is likely that these neurons are glutamatergic since it was recently shown that 85% of the Fos neurons localized in the SLD in the PSR condition are glutamatergic [78]. From these and our results, we propose that the PS-On neurons of the LPGi are turned on at the onset of and during PS by a strong and bilateral excitatory input from the glutamatergic PS-On neurons of the SLD. In turn, the GABA/glycinergic PS-On neurons of the LPGi would participate in the hyperpolarization of i) the facial motoneurons, ii) the LPGi PS-Off neurons and iii) the LC PS-Off noradrenergic neurons.

Our results also indicate that, in addition to the SLD, LPGi neurons are likely controlled during PS by three additional areas, the GiA, the PAG and the lateral hypothalamic area. The DMPAG and GiA contain Fos/CTb neurons only in the PSR condition whereas the LPAG and vlPAG contains such neurons in both PSD and PSR conditions. In the DMPAG and LPAG, the majority of the neurons expressing Fos in the PSR condition also express VGluT2, a marker of glutamatergic neurons (Olivier Clément, personal communication). Interestingly, it has been previously shown that stimulation of the DMPAG, albeit in the anaesthetised animal, evokes excitatory responses in the LPGi [41]. The vlPAG contains a mixed population of Fos/VGluT2 and Fos/GAD67-expressing neurons in the PSR condition whereas most GiA Fos neurons express GAD67 [14]. Taken together, these results suggest that different subdivisions in the PAG control the activity of LPGi neurons during PS, through both glutamatergic and GABAergic neurons.

Finally, neurons clearly identified as PS-On neurons have been recorded in the lateral hypothalamic area [50], [51], with some of them expressing MCH [26] and others the vesicular transporter for GABA [27]. Consistent with this, it has been shown that most MCH and a large number of GAD67 expressing neurons localized in the lateral hypothalamic area are strongly activated during PS hypersomnia [25], [79]. From these and our results it can be proposed that MCH and GABAergic PS-On neurons of the lateral hypothalamic area contribute to the inactivation of the PS-Off neurons of the LPGi during PS.

### Conclusion

**Figure 16:**
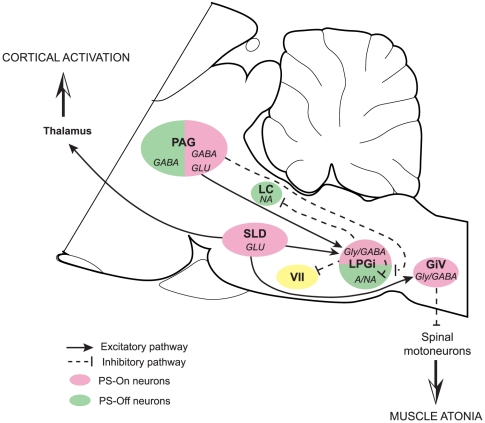
Sagittal representation of the connections of the LPGi activated during PS. During PS, glutamatergic neurons in the SLD strongly excite Gly/GABAergic neurons in the LPGi and the GiV. In turn, Gly/GABAergic neurons of the LPGi contribute to the hyperpolarisation of the facial motoneurons. They also contribute to the inhibition of the local adrenergic/noradrenergic neurons and the distant LC neurons. Glutamatergic neurons in the PAG may excite the LPGi Gly/GABAergic neurons. Moreover, GABAergic neurons in the PAG would inhibit the adrenergic/noradrenergic neurons in the LPGi. Abbreviations: VII: facial nucleus; A/NA: adrenaline/noradrenalin; GiV: gigantocellular reticular nucleus, ventral part; Glu: glutamate; Gly: glycine; LC: locus coeruleus; LPGi: lateral paragigantocellular nucleus; PAG: periaqueductal gray; SLD: sublaterodorsal nucleus.

The LPGi has long been known as a sympathoexcitatory area regulating blood pressure. In this study, we show that this nucleus is an integral part of the network regulating PS through its connections activated by experimental conditions suppressing or increasing the amount of this state of sleep. We propose that, during PS, the LPGi is strongly excited by the SLD, i.e. the pontine executive area of PS, and hyperpolarizes the motoneurons of the facial nucleus and local and LC PS-Off neurons and by this means contributes to PS genesis (Fig. 16).

## Materials and Methods

All experiments were conducted in accordance with the European Community Council Directive (86/609/EEC). The protocols (BH 2006–07 and BH 2006–09) were approved by the Institutional Animal Care and Use Committee of our University (Comité d'expérimentation animale de l'Université Claude Bernard Lyon 1). Every effort was made to minimize the number of animals used and their suffering.

### Electrophysiological study

#### The head restrained rat method

The procedure (chronic implantation for the polygraphic recordings and fixation of the head restraining system) has been previously described in detail [15], [28], [44]. Briefly, male Sprague-Dawley rats (220–240g, Charles River, L'Arbresle, France, n = 30) received an i.p. injection of a mixture of ketamine/xylazine (100 mg/kg and 10 mg/kg, respectively) and were mounted conventionally in a stereotaxic frame (David Kopf, CA, USA). For standard monitoring of the electroencephalogram (EEG), three electrodes were fixed in the skull above the frontal, parietal and occipital cortex and a fourth one was fixed above the cerebellum as a reference. Two wire electrodes were inserted into the neck muscles to monitor the electromyogram (EMG). The head restraining system was then put in place. For this, a U-shaped piece, fixed to a flexible carriage (GFG Co, Pierre-Bénite, France) fastened to the stereotaxic apparatus, was positioned over the LPGi. During the implantation, lidocaine (21 mg/mL) was regularly deposited locally over the lips of the wound. After the implantation, paracetamol (120 mg/kg) was orally administered. After a 2-day recovery, the rats were progressively habituated to the restraining and recording system for 8–10 days. At the end of the training, they could stay calm for 5–6 hours during which quiet W (without movements), SWS and PS were routinely observed. After the habituation process and before the first single-unit recording session, the rats were anesthetized with ketamine/xylazine and a 4 mm hole was drilled over the LPGi. Daily recording sessions were typically performed over a maximum of 7–10 days, each session lasting 4–6 hours. The brain surface was cleaned under local lidocaine anaesthesia at the beginning of each recording session.

#### Recording and analysis of neuronal activity

Extracellular recordings of LPGi neurons were performed using a single-barrel glass micropipette (external tip diameter, 2–3 µm) filled with 0.5M sodium acetate or a neuroanatomical tracer (CTb or PHA-L). Electrode impedance measured at 10 Hz ranged between 5 and 10 MΩ. Filtered (AC, 0.3–10 kHz) and unfiltered (DC) electrode signal were amplified (P16, Grass Instruments, IR USA) and fed to storage oscilloscopes and an audio monitor (GRASS AM8 Audiomonitor). Single unit activity (signal-to-noise ratio of at least 3:1) was isolated with an amplitude spike discriminator (Neurolog Spike Trigger, Digitimer Ltd., UK). The polygraphic signals (EEG and EMG; sample rate 500 Hz each) were amplified (P15, Grass) and filtered (bandwidth of 0.1–100kHz for the EEG and 1Hz-1kHz for the EMG). The single unit activity, polygraphic signal, the AC trace (sampling rate 15 kHz) and a video acquisition of the rat behaviour were simultaneously visualized online during the experiments and stored via a Cambridge Electronic Design (Cambridge, UK) interface using Spike 2 software.

When lowering the recording electrode through the brain, different neuronal activities encountered served as landmarks. First, cerebellar cells were recorded. Purkinje cells with a phasic and irregular discharge pattern were distinguished from granular cells showing a phasic and regular firing pattern. The entrance in the medulla was considered to be located after the last cerebellar cells. About 7–8.5 mm deeper, neurons displaying a typical bursty pattern, strictly in phase with the respiratory movements of the rat, were recorded over a 200–300 µm distance. These neurons belonged to the Bötzinger or pre-Bötzinger nuclei. The neurons recorded after the last respiratory neuron until the ventral surface of the brain, i.e. over a distance of 300–400 µm, were considered as belonging to the LPGi. But it was only after completion of the immunohistochemical reaction aimed at localizing the injection sites of the anatomical tracers that the recording sites were considered as valid. The study presented here is based on recording and injection sites restricted to the part of the LPGi situated caudally to the facial nucleus (11.8 to 13.0 mm posterior to Bregma).

The mean discharge rates of individual neurons recorded during W, SWS and PS were calculated by averaging spike counts made for at least 3 episodes of 10 seconds of recording in one given vigilance state. The effects of behavioural states on the discharge of each class of recorded neurons were assessed with a Friedman test followed by a Wilcoxon's test (paired data). The discharge pattern of neurons was appreciated by first-order interspike interval (ISI) histograms (ISHs), displaying the distribution of intervals between consecutive spikes. For each vigilance state, an asymmetric index was defined as the ratio mode (the most frequent ISI) to the mean ISI (the reciprocal of the mean firing rate). Thus, an asymmetry index near the unit reveals a relatively regular discharge pattern, whereas the more the index differs from the unit, the more irregular is the spike train [80]. The coefficient of variation of firing interval was calculated during all vigilance states. It is defined as the ratio of the standard deviation of ISI to the mean of inter-spikes intervals. Consequently, a bursty neuron is characterised by a high coefficient of variation [49]. The asymmetry index and coefficient of variation were analyzed with a Friedman test followed by a Wilcoxon's test (paired data). The firing pattern of the PS-On neurons was further analyzed by performing a 1-ms bins autocorrelogram using NeuroExplorer software. This method is used to detect periodicities in the spike trains.

Finally, to determine whether the discharge of the neurons considered as PS-On neurons anticipated the onset of PS, the number of spikes was calculated for each neuron and for periods of 10 s covering 60 s before and 60 s after the onset of a PS episode. The onset of PS was set at the point when the muscle atonia showed no further decrease and the EEG simultaneously displayed a low amplitude and high frequency. The end of the episode was always characterized by bursts of activity in the EMG, signalling neck movements and waking of the animal. The initiation of the activation of the individual LPGi neurons was defined as the 10-s period when the number of spikes was at least 2 S.E.M. larger than the value of the previous 10-s.

All data are expressed as mean ± S.E.M. and the significant level for all statistical analyses is set at p≤0.05.

### Neuroanatomical study

#### CTb and PHA-L injections into the LPGi

All the animals used for the electrophysiological experiments received tracer injections, either one tracer only or successively CTb and PHA-L. The injections were usually made after several recording sessions when neurons were consistently kept over a complete sleep-wake cycle. Additional animals (male Sprague-Dawley rats, 220–240g, n = 16) were only used for the anatomical study. In this case, they were anesthetized with ketamine/xylazine and mounted conventionally in a stereotaxic frame with ear bars and head holder. The bone was exposed and cleaned and a hole was made under the LPGi stereotaxic coordinates. A single-barrel glass micropipette (external tip 2–3 µm) filled with CTb or PHA-L was lowered into the brain with a micromanipulator. The localisation of the LPGi was assessed by recording the neuronal activity along the electrode tract, using the same criteria as during the electrophysiological experiments. Once the electrode tip was in place in what was considered as the LPGi, CTb or PHA-L were iontophoretically injected (+2 µA, 7s on, 7s off, during 15 min for CTb; +5 µA during 15 min for PHA-L). After the injection, the electrode was left in place for 10 min. To be able to monitor the sleep-waking cycle during the rest of the experiment, electrodes were fixed in the skull and the neck of the animals, as described above. The animals were allowed a week recovery from surgery in individual Plexiglas containers.

#### Paradoxical sleep deprivation and recovery

PS deprivation was performed using the flowerpot technique that has been previously shown to induce a selective deprivation of PS in rats [32], [81]. This technique is known to cause no significant change in adrenal gland weights, a measure of stress level in animals [81]. Rats were divided into 3 groups: control (PSC), deprived of PS (PSD) and PS recovery (PSR). The PSC animals remained on a bed of woodchips in the recording room throughout the experiment. After 24h of baseline recording and at 10.00 AM, the PSD and PSR rats were placed on a platform surrounded by 2cm of warm water. The platform was just large enough (6.5 cm in diameter) to hold the animal. In this setting, the animal could engage in SWS but not PS because of the loss of muscle tone that occurs during PS. Each morning (between 10.00 and 11.00 AM), the rats were removed from their platform in order to clean the jar and placed in another Plexiglas jar where they could groom and stretch. On the last day, i.e. after 72h of deprivation, the PSR animals were removed from the platform and returned to a dry bed of woodchips in their recording jars to allow a PS recovery for exactly 150 min after the first PS episode (generally occurring after 30–45 min of exploration and grooming). During that time, the PSD rats were kept deprived on the platform. Finally, the PSC animals were first anesthetized for perfusion (at 11.00AM), followed by the PSD and the PSR rats. The 150 min corresponded to the delay required for the maximal production of Fos protein induced by PS hypersomnia [82], [83]. Although Fos is not a perfect indicator of increased spiking activity in neurons [84], it has been recognized as a useful marker of neuronal activation [85] and largely used in the identification of neurons belonging to the PS network. Indeed, several areas displaying a number of Fos-immunoreactive neurons positively correlated to PS quantities during PS hypersomnia were identified by other approaches as playing a critical role in PS genesis (see [3].

#### Sleep scoring and analysis

For each rat included in the anatomical study the determination of the vigilance states was performed offline on the 24-h baseline recording and on the 150 min preceding euthanasia. The vigilance states were scored from 5-sec epochs as W, SWS or PS by visual examination of polygraphic signals and the help of a sliding window showing the EEG power spectrum analyzed during the same 5-sec epochs by fast Fourier transform. Hypnograms were drawn using a custom script in Spike-2. The values were finally exported to Microsoft Excel to calculate the quantities and percentages of each state.

#### Histology and immunohistochemical procedure

The animals were perfused with a Ringer-lactate solution containing 0.1% heparin, followed by 500mL of a fixative composed of 4% paraformaldehyde in 0.1M phosphate buffer (PB, pH 7.4). The brains were postfixed overnight in the same fixative at 4°C and then stored at 4°C for at least 3 days in 0.1M PB with 30% sucrose. They were rapidly frozen in a −50°C isopentane solution. Coronal 25 µm-thick sections were obtained with a cryostat (Microm, France) and stored in 0.1M PB, pH 7.4, containing 0.9% NaCl, 0.3% Triton X-100 (PBST) and 0.1% sodium azide (PBST-Az).

The different antibodies used are listed in Table 4. In addition to the PHA-L single immunostaining and the double Fos/CTb staining, the injection sites were localized in relation to the C1 adrenergic group and the neighbouring cholinergic neurons using tyrosine hydroxylase and choline acetyltransferase antibodies, respectively.

**Table:** **Table 4.** Antibodies used in the immunohistochemical study.

	Fos	CTb	PHA-L	TH	ChAT
Primary antibodies	Rabbit	Goat	Rabbit	Rabbit	Rabbit
Suppliers	Oncogene, USA	List Biological Labs, USA	Vector Labs, USA	Jacques Boy, France	Chemicon, USA
Dilution	1 :10,000	1 :40,000	1 :5,000	1 :5,000	1 :2,500
Secondary antibodies	Donkey anti-rabbit	Horse anti-goat	Donkey anti-rabbit	Donkey anti-rabbit	Horse anti-goat
Suppliers	Rockland	Vector Labs	Rockland	Rockland	Vector Labs
Dilution	1 :1,000	1 :1,000	1 :1,000	1 :1,000	1 :1,000

ChAT: choline acetyltransferase, CTb: cholera toxin subunit B, PHA-L: *Phaseolus vulgaris* leucoagglutinin, TH: tyrosine hydroxylase.

Free-floating sections were successively incubated 1) for 3 days at 4°C in the primary antibody corresponding to the antigen visualized in blue-black in the end of the reaction, e.g. Fos in the Fos/CTb double labeling; 2) 90 min at room temperature in the appropriate biotinylated secondary antibody diluted 1∶1,000 and 3) 90 min at room temperature in an avidin-biotin-HRP complex (Vector Labs) diluted 1∶1,000. To visualize the reaction, the sections were immersed for about 10 min at room temperature in a 0.05M Tris-HCl buffer (pH 7.6) containing 0.025% 3,3′-diaminobenzidine-4HCl (DAB; Sigma, Saint Quentin Fallavier, France), 0.003% H_2_O_2_ and 0.6% nickel ammonium sulphate. When judged optimal under the microscope, the reaction was stopped by two rinses in PBST-Az. If the sections were intended to be processed for a second labeling, they were rinsed overnight in PBST-Az, then incubated 1) for 3 days at 4°C in the primary antibody corresponding to the antigen visualized in brown, e.g. CTb in the Fos/CTb double labeling; 2) 90 min at room temperature in the appropriate biotinylated secondary antibody diluted 1∶1,000 and 3) 90 min at room temperature in the avidin-biotin-HRP complex (Vector Labs) diluted 1∶1,000. The visualization of the reaction was performed as above except that the nickel salt was omitted from the DAB solution. Two rinses of 30 min each were performed between all incubations. At the end of the immunohistochemical reactions, the sections were mounted on gelatin-coated slides, dried, dehydrated and coverslipped with DePex.

The antiserum to Fos was made in rabbit against a synthetic peptide corresponding to the N-terminal part (residues 4-17) of human Fos. This part of the protein displays 100% homology between human and rat and no homology with Fos-related antigens such as Fos B, Jun B, Fra-1 and Fra-2 (Blast 2 sequences, NCBI). The antiserum to CTb was made in goat against the purified B subunit of cholera toxin. The antiserum to PHA-L was generated in rabbit against both the erythroagglutinin and the leucoagglutinin forms of *Phaseolus vulgaris.* The antiserum to tyrosine hydroxylase was made in rat against enzyme isolated and purified from rat pheochromocytom [86]. The antiserum to choline acetyltransferase was made in goat against human placental enzyme.

#### Analysis of the PHA-L labelling

The axons issued from the LPGi were observed in 11 rats and the analysis was only qualitative. The sections from one rat were double-stained for catecholamine neurons with the TH antibody.

#### Cell counts and analysis of the Fos/CTb labelling

The analysis of the neurons singly or double-labelled after the CTb injections in the LPGi was made on 12 animals, 4 PSC, 4 PSD and 4 PSR, similarly to previous works from our laboratory [12], [13], [14], [16], [25], [87]. To illustrate the distribution of the labeling across the brain and count the neurons, drawings of the double-immunostained sections were made with an image analysis system (Mercator, ExploraNova, La Rochelle, France) coupled to a Zeiss Axioskop microscope equipped with a motorized X-Y sensitive stage. Only the single CTb and the double-labelled Fos/CTb neurons were plotted and, once plotted, were automatically counted by the software Mercator. The distribution and numbers of the single Fos neurons, in the areas of interest here, were similar to those observed in previous studies from our laboratory with rats submitted to the same experimental protocol [12], [13], [14], [16], [25], [87]. Consequently, these single Fos neurons were not quantified in the present study. The single CTb and double-labelled Fos/CTb neurons were plotted on sections taken at 600-µm intervals from the caudal level of the LPGi (Bregma -13,30 mm) to the prefrontal cortex (Bregma +2,30 mm), i.e. on 27 sections. For each nucleus of interest, the mean ± SEM number of neurons was determined ipsilaterally and contralaterally to the CTb injection. The nuclei located on the midline were analyzed as a single entity. Most of the neuronal groups being present in more than one section, the neurons counted across the same neuronal group were summed. In all, 40 neuronal groups were analyzed.

For each structure, a Kruskal-Wallis test was performed, with group (PSC, PSD, and PSR) as a factor in order to test the differences in the number of labelled neurons between the experimental groups. Then, a Mann and Whitney test was used to identify significant pairwise differences. The significant level for all statistical analyses was set at p≤0.05.

All photographs were taken with the CCD Color 10-bit QiCam camera used to plot the labelled neurons. They were imported into Adobe Photoshop 7.0, digitally adjusted for brightness and contrast, and were assembled into plates at a resolution of 300 dpi.
